# Full-Spectrum Targeted Mutagenesis in Plant and Animal Cells

**DOI:** 10.3390/ijms22020857

**Published:** 2021-01-16

**Authors:** Brian Iaffaldano, Jakob Reiser

**Affiliations:** Division of Cellular and Gene Therapies, Center for Biologics Evaluation and Research, US Food and Drug Administration (FDA), Silver Spring, MD 20993, USA; jakob.reiser@fda.hhs.gov

**Keywords:** base editing, CRISPR, directed evolution, hypermutation, targeted mutagenesis

## Abstract

Directed evolution is a powerful approach for protein engineering and functional studies. However, directed evolution outputs from bacterial and yeast systems do not always translate to higher organisms. In situ directed evolution in plant and animal cells has previously been limited by an inability to introduce targeted DNA sequence diversity. New hypermutation tools have emerged that can generate targeted mutations in plant and animal cells, by recruiting mutagenic proteins to defined DNA loci. Progress in this field, such as the development of CRISPR-derived hypermutators, now allows for all DNA nucleotides within user-defined regions to be altered through the recruitment of error-prone DNA polymerases or highly active DNA deaminases. The further engineering of these mutagenesis systems will potentially allow for all transition and transversion substitutions to be generated within user-defined genomic windows. Such targeted full-spectrum mutagenesis tools would provide a powerful platform for evolving antibodies, enzymes, structural proteins and RNAs with specific desired properties in relevant cellular contexts. These tools are expected to benefit many aspects of biological research and, ultimately, clinical applications.

## 1. Introduction

The generation of DNA sequence variations is the foundation of directed evolution [1]. The ability to introduce DNA changes into living systems is a bottleneck that limits the exploration of nucleotide sequence space. This is particularly true in plant and animal cell contexts. Many mutagenesis tools have been developed in bacterial and yeast systems [1]; however, outputs from these systems do not always translate to higher organisms. Moreover, many of the methods available result in genome-wide mutations that can reduce cell viability and, therefore, confound experiments and limit the number of mutations that can be introduced at a particular locus. Targeted mutagenesis through the localization of mutagenic proteins is facilitated by emerging tools, such as Clustered Regularly Interspaced Short Palindromic Repeats (CRISPR)/CRISPR-associated protein (Cas)-derived DNA binding domains that can generate precise mutations in cells, while concentrating mutations in targeted regions. With the ability to easily multiplex guide RNAs (gRNAs), CRISPR can simultaneously target many defined regions [2,3,4]. New hypermutation tools hold promise for programmable DNA diversification in plant and animal cells, where all base conversions are possible.

## 2. Directed Mutagenesis Using CRISPR Nucleases

Directed mutagenesis can be achieved using CRISPR nucleases to target certain genomic regions, or gRNA libraries can be used to target regions throughout the genome. This has been successfully demonstrated in yeast [5], mammalian cells [6] and plants [7]; however, the majority of mutations introduced by CRISPR/Cas nucleases result in small and sometimes larger insertions and deletions (indels), frequently causing frameshift mutations and introducing premature stop codons, while substitutions are rare. With a large proportion of mutations being loss-of-function mutations, this approach is typically not suitable for protein evolution. In addition, edits by CRISPR nucleases are typically restricted to the site of DNA cleavage. Double-stranded breaks can be used to introduce diversity through homology-directed repair (HDR), by co-transfecting an oligo template library, which can contain prescribed indels and point mutations [8]. In yeast, gRNA expression cassettes have been co-introduced with donor libraries to conduct functional analyses of target genes [5]. The same strategy was also used to target hundreds of open reading frames (ORFs) across the entire yeast genome [5]. In mammalian cells, single guide RNAs (sgRNAs) coupled with a donor library approach have been adapted for in vivo antibody engineering [8]. A similar approach has been used for in situ fluorescent protein engineering to evolve pH resistance, allowing for fluorescence in particular subcellular environments [9]. However, the efficiency of precise genome editing by HDR remains low in many biological systems, and most of the double-strand breaks (DSBs) are repaired through the non-homologous end joining (NHEJ) pathway, even when an abundance of repair donor templates is present. For instance, the HDR editing efficiencies in plants are usually below 10% [10]. Moreover, the editing efficiency is negatively related to the distance between the DSB site and the designed mutations, increasing the difficulties in introducing diversity [5]. In addition, the breadth of diversification is dependent on the size and design of the donor library, which can increase cost and labor requirements. Overall, directed mutagenesis approaches using CRISPR/Cas nucleases suffer from undesirable frameshift mutations and are limited in the amount of sequence space that can be explored.

## 3. Diversifying Base Editors

**Figure 1:**
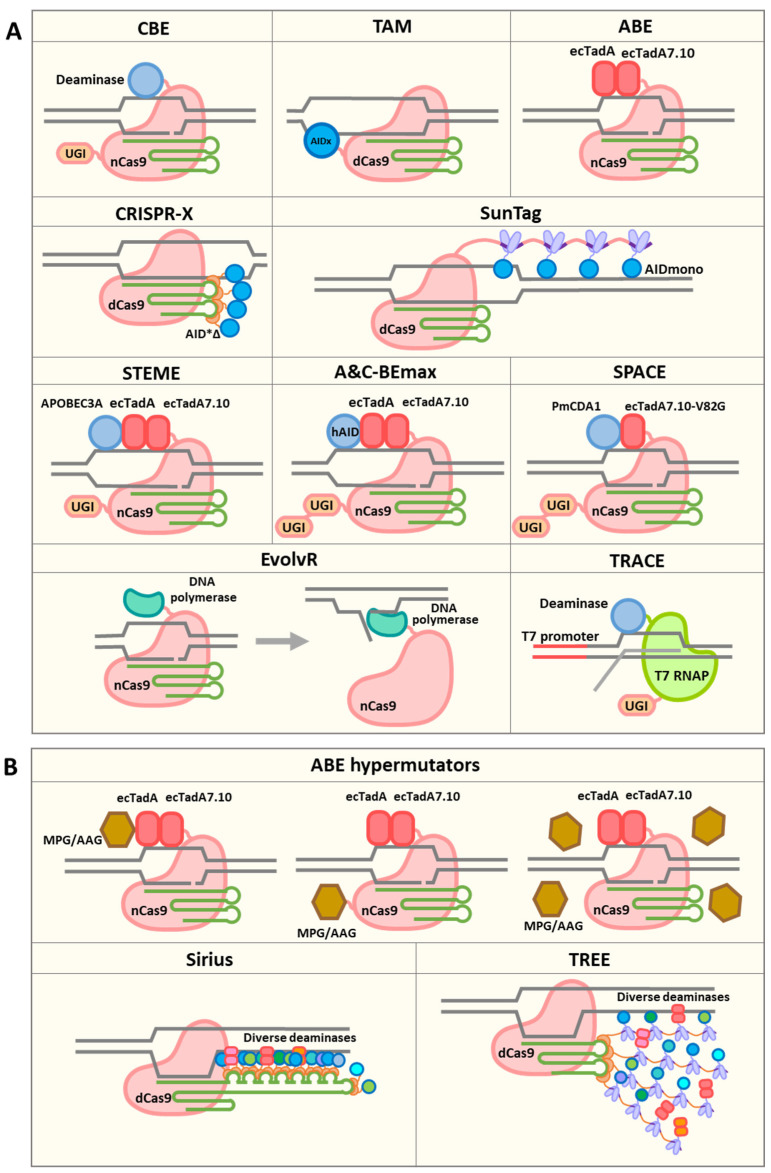
Available and hypothetical CRISPR-derived hypermutators and TRACE system. (**A**) CRISPR-derived hypermutators and TRACE system. CBE is used to achieve C-to-T conversions. CBE-derived hypermutators include TAM, CRISPR-X and the SunTag-based hypermutator. ABE is used to achieve A-to-G conversions. Dual cytosine and adenine base editor systems include STEME, A&C-BEmax and SPACE. EvolvR uses a Cas9 nickase and an error-prone, nick-translating DNA polymerase to achieve targeted mutagenesis. TRACE uses a T7 RNA polymerase fused to a cytidine deaminase to introduce mutations in genes driven by a T7 promoter. (**B**) Hypothetical CRISPR-derived hypermutators. ABE-derived hypermutators can be developed by recruiting MPG/AAG to the target sites or overexpressing MPG/AAG. Other systems, including Sirius and TREE, can be used to recruit diverse deaminases to achieve full-spectrum mutagenesis.

Base editing uses CRISPR to recruit deaminase proteins, which can introduce base substitutions in defined genomic regions [11]. Upon the formation of Cas–gRNA–DNA R-loops, single-stranded DNA (ssDNA) in the R-loop can serve as the substrate for deaminases. DNA bases can be deaminated and fixed through DNA repair or replication. Base editors can be used to generate mutations with minimal indel formation, allowing diversity to be introduced, without reducing the effective population size by causing frameshift mutations (Figure 1A). The first developed base editor, base editor 1 (BE1), consists of a catalytically dead Cas9 (dCas9) protein fused with a cytidine deaminase enzyme, rat apolipoprotein B mRNA editing enzyme catalytic polypeptide 1 (rAPOBEC1) [11]. BE1 targets ssDNA and deaminates the exocyclic amine group on cytosine (C), resulting in a C-to-uracil (U) transition [11]. However, upon the formation of a G–U mismatch, uracil N-glycosylase (UNG) will usually initiate base excision repair (BER), leading to the reversion of the base edit [12]. To improve editing efficiency, the second-generation base editor, BE2, included a uracil glycosylase inhibitor (UGI) fused to dCas9 to inhibit UNG, resulting in efficient C-to-T and G-to-A base editing [11]. The third-generation base editor (BE3) replaced dCas9 with a Cas9 nickase (nCas9) to nick the DNA strand; as a result, repair favors using the edited strand as a template, which further improves editing efficiency [11]. However, by design, these base editors all have narrow editing windows of about 5 bp within the protospacer sequence [11,13]. Several cytosine base editors (CBE) have subsequently been developed, utilizing various deaminases and Cas systems. For example, the Target-AID system utilizes a *Petromyzon marinus* cytidine deaminase 1 (PmCDA1) and exhibits a slightly shifted, but similarly narrow, editing window [14,15]. Interestingly, the replacement of the *Streptococcus pyogenes Cas9* (SpCas9) nickase with a *S. aureus* Cas9 (SaCas9) nickase enlarged the editing window to 10 bp [16,17]. Cas12a-derived base editors also have very narrow editing windows [18]. On the other hand, adenine base editors (ABEs) have been engineered to deaminate the exocyclic amine group on adenosine to form inosine [19]. Inosine usually pairs with guanosine during DNA replication; thus, A-to-C and T-to-G transitions can be achieved. The *E. coli* tRNA adenosine deaminase enzyme, TadA, was evolved to accept ssDNA as the substrate. This evolved TadA* monomer can interact with the wild-type non-catalytic TadA monomer and form a heterodimer. Fusing this heterodimer with a Cas9 nickase can achieve targeted A-to-C and T-to-G base editing [19]. The editing windows of ABE7s are usually about 3–5 bp with SpCas9 and 7 bp with SaCas9 [19,20,21]. The further engineered ABE8s have an overall improved editing efficiency and higher activity outside the 3–5 bp editing window. ABE8s without the wild-type TadA monomer show similar performance to those that possess the wild-type TadA [22]. However, the narrow editing windows of cytidine and adenine base editors limit their potential for broad sequence diversification.

To leverage the easily programmable nature of CRISPR nucleases for diversification, efforts have been made to engineer diversifying base editors. A major defining feature of a base editor is the deaminase, which dictates the mutation frequency and typically targets a preferred hotspot motif (Table 1). Two DNA diversification systems have been developed (Figure 1A). One system, termed Targeted AID-mediated mutagenesis (TAM), utilizes dCas9 with a hyperactive, activation-induced cytidine deaminase P182X (hAIDx) fused to the C terminus [23]. AID can induce somatic hypermutation during antibody maturation and is often used as a cytidine deaminase [24,25]. The dCas9 with an inactivated nuclease domain is used as an RNA-guided DNA-targeting moiety to recruit the associated deaminase domain to target loci [26]. TAM exhibits substitution frequencies greater than 0.4 nt per kb per cell cycle [23]. A similar strategy utilizing dCas9 with a hyperactive AID mutant (AID*Δ), termed CRISPR-X, has also been used [27]. Notably, CRISPR-X does not localize deaminase domains through protein fusion; rather, it uses additional RNA loops derived from the bacteriophage MS2 genome (MS2 loops) within the gRNA sequence to recruit as many as four MS2 coat protein domains fused with deaminases. This may contribute to its broader diversification window and higher mutagenesis activity of ~1–2 nt per kb [27]. To further improve mutation efficiency, a stabilized monomeric AID mutant, referred to as the AIDmono mutant, has been used in the same configuration as CRISPR-X, and higher mutation frequencies were observed (Figure 1A) [15].

An essential characteristic of diversifying base editors is the editing window. The editing window depends not only on the activity of the deaminase, but also on the location of the deaminase domain. When the deaminase is directly fused to dCas9, the editing window is typically limited to the protospacer sequence [23]. The use of sgRNAs with two MS2 RNA aptamers to recruit four molecules of AID in the CRISPR-X system allowed the expansion of the mutation window to about 100 bp surrounding the sgRNA protospacer adjacent motif (PAM) sequence (Figure 1A) [27]. Similar results have been observed using AIDmono [15]. When further increasing the number of recruited deaminases, by, for example, using the SunTag system to recruit 24 deaminase domains, the editing window was not further expanded [15], possibly due to steric interference caused by the use of large recruitment proteins (Figure 1A). When larger genomic regions need to be diversified, multiple sgRNAs can be used simultaneously. It has been observed in the TAM system that the editing window is enlarged when using multiple sgRNAs, possibly due to the increased accessibility of ssDNA [23]. Multiple sgRNAs can be delivered into cells in one construct using diverse multiplexing strategies, including using native RNA processing systems (e.g., RNase III and Cas12a), using self-cleaving tRNAs and ribozymes, and using exogenous RNA-processing enzymes (e.g., Csy4) [3,28]. Larger numbers of sgRNAs can be introduced into cells using a CRISPR library [29,30]. Pooled sgRNAs can be cloned into expression vectors and used to target large DNA regions.

Deaminases play an important role in the footprint of base conversions observed. It has been demonstrated that deaminases retain their intrinsic nucleotide preferences when used in the context of CRISPR-mediated diversifying base editors [15]. AID and its variants, as well as PmCDA1, all have a preference for mutating the C nucleotide in WRC motifs (W = A or T; R = A or G) [15,23]. By contrast, the hotspots for rAPOBEC1, APOBEC3A and APOBEC3B mutant (A3BAct) are TCA and TC [15]. To expand the editing footprint of AIDmono, the substrate recognition loops of APOBEC deaminases have been grafted to AIDmono, resulting in high-activity deaminases with unique mutagenesis hotspots [15]. The hotspots of all the mentioned deaminases are summarized in Table 1. It is also notable that targeted genes can be codon optimized to increase the presence of mutagenesis hotspots throughout or at regions of interest [15].

The deaminases used for DNA diversification are typically cytidine deaminases. They convert C (G on the opposite strand) to all other bases, with a slight preference for T (A on the opposite strand) [15]. This is because the deaminated cytosine can be read as thymine; alternatively, the damaged base can be excised by uracil glycosylase, resulting in a full spectrum of substitutions [12]. While with the inclusion of UGI, an inhibitor of the key enzyme UNG in the base excision repair pathway, editing efficiency can be improved, this largely restricts editing outcomes to C-to-T and G-to-A changes [11,15]. Similarly, in UNG and MutS homolog 2 (MSH2) double-knockout mutant cells, the total mutation rate was increased, but the editing purity was also increased [15], an outcome that is not desirable in diversification applications. In addition, the use of nCas9 can increase the editing efficiency inside the protospacer sequence by reducing the use of the unedited strand as a repair template, but it also restricts the editing window and introduces more indels than dCas9-based strategies [15,23]. This reduction in the hypermutation footprint may be caused by the dissociation of the Cas9 complex from the DNA after the nick is introduced. This more-localized mutagenesis outcome with a higher potential for frameshift mutations may be desirable for evolving structural RNAs or proteins with multiple reading frames.

## 4. EvolvR

In addition to deaminase-derived base editors, directed mutagenesis can also be achieved by using engineered low-fidelity DNA polymerases. In the EvolvR system, nCas9 is fused to an error-prone, nick-translating DNA polymerase (Figure 1A) [31]. When nCas9 generates a nick at the target site, the polymerase initiates DNA synthesis downstream of the nick, and the original nucleotides form a flap that is ultimately replaced. By fusing nCas9 with DNA polymerases of varying fidelity, the mutation rate can be modulated. Moreover, the mutagenesis windows and substitution bias can be fine-tuned by using polymerases with variable processivity and misincorporation bias. Halperin et al. were able to achieve mutation rates 7,770,000-fold greater than baseline and observed editing windows of up to 350 nucleotides from the nick site [31]. Although EvolvR has only been demonstrated to work in *E. coli* to date, it provides a novel avenue for achieving targeted mutagenesis via the CRISPR system, which may ultimately be used in plant and animal cells.

## 5. Other Approaches for Targeted Mutagenesis

While CRISPR/Cas systems are opening new avenues for hypermutation, they are not requisite. One novel approach for targeted hypermutation is T7 polymerase-driven continuous editing (TRACE), which uses a T7 RNA polymerase fused to a cytidine deaminase to introduce mutations in gene sequences driven by a T7 promoter (Figure 1A) [32,33]. As the gene of interest is uniquely driven by a T7 promoter, the targeted gene is predominantly deaminated. T7 RNA polymerase moves along the entire gene sequence during transcription, separating DNA strands and making ssDNA accessible for deamination. In human cells, TRACE was able to generate C-to-T and G-to-A nucleotide substitutions with a frequency ranging from ~0.5 to 4 nt per kb at genomic regions in a window of 2000 bp within a week of expression [32]. TRACE provides a unique method for generating mutations at a high rate within large transcribed regions.

## 6. In Situ and Ex Vivo Directed Evolution Using Targeted Mutagenesis

Targeted mutagenesis using hypermutators has recently been used for the directed evolution of proteins (Table 2). In mammalian cells, one emerging usage is for antibody engineering. Liu et al. used AIDmono- and APOBEC3A-derived hypermutators with the CRISPR-X configuration to evolve the anti-hapten 4-hydroxy-3-nitrophenyl acetyl antibody B1-8, to improve antigen affinity. They identified established mutations that increase antibody affinity and also discovered novel mutations that improve binding affinity [15]. Similarly, Devilder et al. achieved the ex vivo evolution of a low-affinity human monoclonal antibody A2Ab using CRISPR-X. A combination of five mutations were identified, which increased the antibody affinity by nearly two logs compared to the original antibody [34]. Another application of hypermutators is introducing drug resistance in cells. EvolvR has been successfully used to target the *E.coli rpsE* and *rpsL* genes encoding the 30S ribosomal proteins S5 and S12, respectively, resulting in mutations conferring spectinomycin and streptomycin resistance, respectively. In addition, simultaneously targeting both genes yielded results similar to those when targeting each gene individually, suggesting the potential for CRISPR-derived hypermutators to evolve multiple genes or loci simultaneously [31]. Ma et al. used the TAM system to identify mutations that confer resistance to imatinib (Gleevec) in human K562 cells. Such mutations limit the efficacy of imatinib therapy for treating chronic myelogenous leukemia (CML). Multiple imatinib-resistance mutations were identified, including one that was previously found in CML patients, as well as novel mutations that confer resistance to a variety of imatinib doses [23]. TRACE has been used to target mitogen-activated protein kinase kinase 1 (MEK1 kinase) in human A375 cells to screen for mutations conferring resistance to the inhibitors selumetinib and trametinib. This screening identified two novel mutations [32]. In plants, a broad spectrum of targeted mutagenesis has allowed the discovery of novel sequences conferring herbicide resistance. Li et al. conducted directed evolution by diversifying the rice *acetyl-coenzyme A carboxylase* gene (*OsACC*) using the STEMES system and selecting with the ACC inhibitor haloxyfop, which is commonly used as an herbicide. In addition to discovering novel herbicide-resistant mutations, known mutations were also reaffirmed [35]. In addition, Chen et al. successfully introduced one mutation using TRACE that can shift the fluorescence spectrum of blue fluorescent protein (BFP) to that of green fluorescent protein (GFP) [32]. To date, most directed evolution studies in mammalian and plant systems are still in the proof-of concept phase. However, the further development of hypermutators and evolution strategies will likely enable the exploration of more sequence space and allow the selection of sequences with more complex phenotypes.

## 7. Limitations and Future Prospects

To date, CRISPR-mediated directed mutagenesis has been limited due to the NGG PAM requirement of Cas9. The number of sgRNAs that can be designed in the vicinity of certain genomic regions is low. To broaden the accessibility of DNA sequences for the CRISPR system, Cas9 variants and orthologs, as well as other Cas proteins, can be used to recognize diverse PAM sequences [28,36,37,38]. For instance, xCas9 can recognize NG, GAA and GAT PAMs [39], while SpCas9-NG can recognize relaxed NG PAMs [40]. The PAM requirement for SaCas9 is NNGRRT [41], while for the engineered SaCas9 KKH variant, it is NNNRRT [42]. Furthermore, Cas12a and Cas12b recognize thymine-rich PAMs, providing user-friendly platforms for targeting AT-rich regions [43,44]. Recently, a further evolved SpCas9 variant, SpRY, has been developed, which can efficiently target NRN PAMs [45]. The use of multiple, orthogonal CRISPR systems offers the potential to evolve Cas proteins ex vivo, where one CRISPR system diversifies another, which is then screened for desirable characteristics. Notably, SaCas9 and SpCas9 have been demonstrated to operate orthogonally within the same cells [46].

The method used to localize deaminases plays a critical role in determining the editing window. A number of recruiting systems have been used for gene regulation and genomic region illumination, which could potentially be used to recruit deaminases. For instance, the CRISPR-Sirius system can recruit 16 effector proteins through an engineered sgRNA scaffold with eight MS2 aptamers (Figure 1B) [47]. The TREE system, which combines the MS2 and SunTag systems, has the ability to recruit even more effector proteins (Figure 1B) [48]. With more deaminases recruited to genomic regions of interest, the editing window could be enlarged. Notably, deaminases only change nucleotide bases in ssDNA. The access of deaminases to ssDNA is the key factor affecting the editing windows. With the ability to recruit a greater number of deaminase copies, the mutation efficiency might also be improved. Another advantage of using high-capacity recruiting systems is that multiple types of deaminases can be recruited simultaneously, potentially allowing all nucleotides to be targeted in a window and allowing hotspot motifs to complement each other, resulting in a more even distribution of mutations (Figure 1B). 

Thus far, diversifying base editors have used cytidine deaminases, lacking the ability to meaningfully diversify A and T nucleotides. This limits the gene space and codons that can be explored. However, ABEs can deaminate adenines to inosines (I), which are read as guanine (G) by DNA polymerases, resulting in highly efficient A-to-G or T-to-C substitutions [19,22]. Recently, three dual cytosine and adenine base editor systems, including saturated targeted endogenous mutagenesis editor (STEME) [35], A&C-BEmax [49] and synchronous programmable adenine and cytosine editor (SPACE) [50], have been developed in plant and mammalian cells (Figure 1A). By fusing deaminases from CBE and ABE to nCas9 or nCas9-NG, high-frequency C-to-T and A-to-G substitutions have been achieved. 

One limitation of using ABEs for diversification is their high product purity. The edits introduced by ABEs are overwhelmingly A-to-G conversions (~99.9%), while conversions to T or C are rare. In addition, similarly to CBEs, ABEs exhibit narrow editing windows [19]. Therefore, to develop diversifying base editors using ABEs, new deaminase and recruitment strategies with wider editing windows need to be explored. Moreover, the diversification potential might be improved by manipulating DNA repair pathways. Attempts have been made to increase ABE editing efficiency by fusing inactive enzymes involved in inosine binding (human alkyl adenine DNA glycosylase, AAG) or removal (*E. coli* Endonuclease V) to block excision by endogenous proteins, similarly to how UGIs are used in CBEs to limit base excision and increase editing efficiency and product purity [19]. However, these approaches did not improve editing efficiencies. Given the high editing purity of ABEs, this suggests that the excision of inosine is a highly inefficient process, at least in the context of the cell types that have been tested thus far. 

The literature suggests avenues to increase the potential for adenine deaminases to introduce sequence diversity, by boosting inosine excision [19]. Hypoxanthine generated by the spontaneous deamination of adenine in cells can be removed through the base excision repair pathway. This pathway is initiated by N-methylpurine glycosylase (MPG), also known as alkyl adenine glycosylase (AAG), generating an apurinic/apyrimidinic (AP) site. Translesion synthesis (TLS) can occur over the AP site and produce random nucleotides on the opposite strand of DNA, resulting in the conversion of A to all other nucleotides. By recruiting MPG/AAG to the target sites, or overexpressing MPG/AAG through either transient or stable transfection, inosine could be potentially more efficiently converted to all other bases.

In addition, several base editors that can catalyze base transversions have been developed. In *E. coli*, high C-to-A base editing efficiency has been achieved using AID-nCas9-UNG [51]. In mammalian cells, C-to-G base editors (CGBEs) have been developed by fusing rAPOBEC1 and UNG to nCas9 [51,52]. Kurt et al. found that C-to-G editing efficiencies were improved when introducing an R33A change into rAPOBEC1, while removing UNG had minimal effects on editing efficiency [52]. A similar study used X-ray repair cross-complementing protein 1 (XRCC1, an enzyme involved in base excision repair) instead of UNG to achieve C-to-G editing [53]. These systems suggest that deaminase mutations can modulate the ratios of base outcomes during hypermutation and may help to yield improved diversifying base editors in the future.

In addition to directed evolution, the aforementioned mutagenesis platforms that can diversify large regions of user-defined sequence could potentially be used for cellular barcoding. Diverse edits can be continuously introduced to DNA sequences, which can later be sequenced to track large populations of cells. One application of cellular barcoding is lineage tracing, which has become increasingly important in stem cell research and developmental biology. CRISPR-Cas9 has been used to create NHEJ-mediated indels in pre-integrated repeat sequences for lineage tracing; systems such as genome editing of synthetic target arrays for lineage tracing (GESTALT) [54] and memory by engineered mutagenesis with optical in situ readout (MEMOIR) [55] have been established. CRISPR lineage tracing can be used simultaneously with single-cell RNA sequencing (scRNA-seq) to identify cell types in different organs and tissues. This has recently been demonstrated in zebrafish [56,57] and mice [58]. Since NHEJ-mediated genome editing results in indels within the target sequence, this will prevent further editing, and gRNA will lose the ability to generate unique barcodes over time. Hwang et al. demonstrated lineage tracing using a base-editing strategy, in which a nCas9 was fused to a cytidine deaminase and targeted to endogenous repetitive L1 elements [59]. This study provides an example of another avenue for generating cellular barcoding using CRISPR with high resolution. In addition to lineage tracing, cellular barcoding can also be used for genetic recording. For instance, Perli et al. developed a CRISPR-based recording system termed mammalian synthetic cellular recorders integrating biological events (mSCRIBE) [60]. When using one or more self-targeting gRNAs and inducible promoters to drive gRNA or Cas9, mSCRIBE successfully recorded the duration and intensity of biological events when cells were exposed to certain stimuli. The hypermutators we have described, including diversifying base editors, EvolvR and TRACE, can generate diverse mutations in a broader genomic window than base editors and CRISPR nuclease-derived diversifying strategies. This may allow such hypermutators to achieve finer resolutions in barcoding for lineage tracing and genetic recording. The use of hypermutators for barcoding experiments is another research avenue yet to be explored. 

## 8. Conclusions

CRISPR-based mutagenesis approaches have significantly expanded the ability to improve ex vivo directed mutagenesis [61]. In the future, novel targeted diversifying strategies can be developed. Coupling with the use of gRNA libraries, CRISPR-mediated directed mutagenesis approaches will provide powerful and high-throughput tools for directed evolution, forward genetics and lineage studies in relevant cellular contexts. The recruitment of both adenine and cytosine base editors with CRISPR or polymerase-based approaches such as EvolvR and TRACE will potentially allow the mutagenesis of all bases within defined sequence windows in plant and animal cell contexts. Future work focusing on boosting the excision of inosine may allow all substitution outcomes to be realized. Targeted hypermutation strategies provide a growing toolbox for conducting genomic barcoding, studying genetics and evolving proteins and RNAs in relevant cell contexts.

## Figures and Tables

**Table 1:** Editing hotspots and profiles of cytidine deaminases used for hypermutation.

Deaminase	Hotspot ^1^	Editing Profile	Editing Profile (With UGI or in UGN/MSH2 Double Mutant)	Reference
hAIDx (P182X)	WGC	C/G to all nucleotides, with a preference for C/G to T/A	C/G to T/A	[15,23]
hAID*Δ	WRC			[15]
AIDmono	WRC, AGCT			
PmCDA1	WRC			
APOBEC3A (A3A)	TCA, TC, TCC (using nCas9)			
APOBEC3B mutant (A3BAct)	TCA, TC			
rAPOBEC1	TC			
AID-3C (AIDmono with APOBEC3C substrate-recognition loop)	TTC			
AID-3F (AIDmono with APOBEC3F substrate-recognition loop)	TGC			

^1^ W = A or T; R = A or G.

**Table 2:** In situ and ex vivo directed evolution using targeted mutagenesis.

Target Protein	Evolution Goal	Evolution System	Biological System	Identified Major Mutations	Reference
Anti-hapten 4-hydroxy-3-nitrophenyl acetyl antibody B1-8	Improve antigen affinity	CRISPR-X with AIDmono/APOBEC3A	HEK293T cells	W33L, T30I, T30S, S31R, T58I, A97G	[15]
Human monoclonal antibody A2Ab	Improve antigen affinity	CRISPR-X with AID*Δ	HEK 293 cells	D74H, W102 L, M112I, G121D, R124P	[34]
30S ribosomal proteins S5 and S12	Spectinomycin and streptomycin resistance	EvolvR	*E. coli*	Δ17–19, K23N and Δ24, Δ24, Δ26, G27D (*rpsE*)	[31]
BCR-ABL	Imatinib (Gleevec) resistance	TAM	Human K562 cells	T315I, T319I	[23]
Mitogen-activated protein kinase kinase 1 (MEK1 kinase)	Selumetinib and trametinib resistance	TRACE	Human A375 cells	E38K, V211D	[32]
Acetyl-coenzyme A carboxylase (ACC)	Herbicide (haloxyfop) resistance	STEMES	Rice	W2125C, P1927F, S1866F, A1884P	[35]
Blue fluorescent protein (BFP)	Shift fluorescence spectrum to that of green fluorescent protein (GFP)	TRACE	HEK293T cells	H66Y	[32]

## Abbreviations

AAG	Alkyl adenine DNA glycosylase
ABE	Adenine base editor
ACC	Acetyl-coenzyme A carboxylase
AID	Activation-induced cytidine deaminase
AIDmono	Monomeric AID
AP site	Apurinic/apyrimidinic site
APOBEC3A	Apolipoprotein B mRNA editing enzyme catalytic polypeptide 3A
APOBEC3B	Apolipoprotein B mRNA editing enzyme catalytic polypeptide 3B
BE	Base editor
BER	Base excision repair
BFP	Blue fluorescent protein
Cas	CRISPR-associated protein
CBE	Cytosine base editor
CGBE	C-to-G base editor
CRISPR	Clustered regularly interspaced short palindromic repeats
dCas9	Catalytically dead Cas9
DSB	Double-strand break
GESTALT	Genome editing of synthetic target arrays for lineage tracing
GFP	Green fluorescent protein
gRNA	Guide RNA
HDR	Homology-directed repair
Indels	Insertions and deletions
MEK1 kinase	Mitogen-activated protein kinase kinase 1
MEMOIR	Memory by engineered mutagenesis with optical in situ readout
MPG	N-methylpurine glycosylase
mSCRIBE	Mammalian synthetic cellular recorders integrating biological events
MSH2	MutS homolog 2
nCas9	Cas9 nickase
NHEJ	Non-homologous end joining
ORF	Open reading frame
PAM	Protospacer adjacent motif
PmCDA1	*Petromyzon marinus* cytidine deaminase 1
rAPOBEC1	Rat apolipoprotein B mRNA editing enzyme catalytic polypeptide 1
scRNA-seq	Single-cell RNA sequencing
sgRNA	Single guide RNA
SPACE	Synchronous programmable adenine and cytosine editor
ssDNA	Single-stranded DNA
STEME	Saturated targeted endogenous mutagenesis editor
TAM	Targeted AID-mediated mutagenesis
TLS	Translesion synthesis
TRACE	T7 polymerase-driven continuous editing
UGI	Uracil glycosylase inhibitor
UNG	Uracil-DNA glycosylase
XRCC1	X-ray repair cross-complementing protein 1
